# The Sydenham Society

**Published:** 1858-03

**Authors:** 


*The Sydenham Society.*—We referred in our last number to the rumored demise of the Sydenham Society, and the project which had been entered upon of forming a new society. We now present from the Boston Journal the latest news in regard to this institution, in which we all have so much interest. It will be seen by the following article, that the author's information comes from the most reliable source: F.

We lately referred to the affairs of this Society, and expressed the hope that the benefits accruing from it, or from a similar organization, would be still continued to the profession. Conversation with the "Local Secretary" here, Dr. Salter, has put us in possession of the facts relative to its present condition and future prospects; and it is nearly certain that not only will the Society be maintained, but that its usefulness will be decidedly increased. The leading British medical journals have something to the same effect in their late issues, and there seems to be a wish to sustain a well-managed association of this kind. To our mind, most of the books which have been published under the old and now defunct administration are valuable, and well deserve a place in all medical libraries. Surely the works of Hippocrates, Sydenham, Harvey, Hewson, Louis, Simon, Wedl, Rokitansky, Kölliker, Romberg, Prochaska, Dupuytren, Velpeau, Kuchenmeister, and others of the list, are such as no medical man would willingly be without. On the whole, we think the Society has been successful and judicious; if it be true that more practical works are *now* desirable, it is no less so that those already presented to the members have a great and permanent value, and that it would be a matter for regret had they not been furnished. Even the Lancet, which has shown a somewhat bitter feeling toward the Sydenham Society from the very first, hardly becoming a journal of so high a tone, whatever be the motives actuating the demonstration, has lately very properly insisted upon the advantages to be derived by medical students and practitioners from a study of the older authors, and has named, amongst such, several of the writings supplied by the Society of which it has professed to think so slightly. It were indeed well did the members of our profession, and especially the younger men, oftener turn the leaves of those rare old works which "young Physic" is too apt to term musty and dry. Instead of merely skimming the surface of the periodical literature of our art, and trusting solely to Braithwaite and Ranking, let them oftener ponder the Fathers of Medicine; it will be a more lasting "*retrospect*," and they will derive from them a more effective "*abstract*." Let us not be understood as under rating the above named valuable collections of medical facts and opinions; there is no need to neglect *them*, but a place may be fitly given to the others. No reader, and especially no student, can rise unimproved from a perusal of Sydenham; and we do not believe the most progressive practitioner would be injured by a peep into the volumes of old Paul of Ægina, nor could it harm him to read the remarkable account of the Epidemics of the Middle Ages, by Hecker—for issuing the last work the Sydenham Society has been sneeringly censured. We say then, that whilst such a society should see that the majority of its publications are those which may wholly advance our *practical* knowledge, a sprinkling of such as are more general is highly appropriate. Especially might some which are historical of medical science be wisely chosen. We do not believe that one student—scarcely one practitioner—out of every hundred, knows anything about the *history* of the profession he has adopted; and the same is true for the proportion of them who know anything of the valuable old writings, some of which we have enumerated.

The British and Foreign Medico-Chirurgical Review, for January, 1858, speaks as follows in relation to the Sydenham Society. "That the late Society has conferred a great boon upon the profession of this country, there can, we think, scarcely be a doubt; and while it is very possible that the time had arrived at which its regeneration was desirable, and at which it became necessary that such an association should be guided by somewhat different principles from those upon which the council have hitherto proceeded, we are equally confident that the class of literature which has been rendered accessible to the profession of this country by the agency of the old Sydenham Society, has tended much to elevate the scientific status of its members."

After stating that the old Society often contemplated publishing "more modern and more strictly practical works," than the majority of theirs were, the Review expresses the hope that the difficulties which prevented the execution of such intentions, may be overcome by the new Sydenham Society. A number of gentlemen, it appears, have already met for the purpose of reconstituting themselves into such a Society, and a Prospectus has been offered, designatory of their plan, from which we quote the titles of a few of the volumes proposed: "Marx's "Life of Paracelsus," translated from the German; Hebra's work on Diseases of the Skin, with atlas of plates, &c.; Gooch "On the More Important Diseases of Women;" Pirogoff's "Surgical Essays;" "Diday on Hereditary Syphilis," with annotations; a collection of facts upon Bronzed Skin and Diseases of the Supra-Renal Capsules; Dieffenbach's "Operative Surgery," with annotations; Heberden's "Commentaries;" "The Father's of British Surgery," being selections from the works of Wiseman and others; "Modern Military Surgery," &c., &c.

This list promises well, and we cannot but hope that when the new organization is effected, not only all the old subscribers will remain, but a legion of new ones be added; and we would earnestly advise those who have hitherto been members in the United States, to adhere to their membership; and those who have not, to keep their eyes open for the moment when the new books shall appear, and remember the wisdom of speaking for everything desirable, *in season*. With the Review from which we have extracted the above paragraphs, we cry, "*Le Roi est mort, vive le Roi!*"

